# CLSP-REQA: a real-time quality-aware closed-loop seizure prediction framework with Mamba-BiLSTM and confidence-gated intervention

**DOI:** 10.3389/fninf.2026.1888743

**Published:** 2026-09-30

**Authors:** Mufeng Chen, Qi Wu, Bingchao Huang, Xiwen Lai, Zekai Chen, Xinge Ouyang, Quansheng Ren

**Affiliations:** 1 Department of Engineering Science, University of Oxford, Oxford, United Kingdom; 2 Mathematical Institute, University of Oxford, Oxford, United Kingdom; 3 School of Computer Science and Engineering, Beihang University, Beijing, China; 4 Aerospace Information Research Institute, Chinese Academy of Sciences, Beijing, China; 5 Department of Mechanical Engineering, The University of British Columbia, Vancouver, BC, Canada; 6 College of Life Sciences, Hunan Normal University, Changsha, China; 7 School of Electronics, Peking University, Beijing, China

**Keywords:** bidirectional LSTM, closed-loop neurostimulation, confidence calibration, cross-patient generalization, EEG signal quality assessment, epileptic seizure prediction, Mamba

## Abstract

**Introduction:**

Reliable seizure prediction is a prerequisite for closed-loop neurostimulation therapy, yet existing methods rarely account for the variability in EEG signal quality encountered in real-world deployment, and the overwhelming majority adopt non-strict evaluation protocols that overestimate generalization performance.

**Methods:**

We propose CLSP-REQA (Closed-Loop Seizure Prediction with Real-time EEG Quality Assessment), a unified framework that embeds a lightweight signal quality estimator directly within the prediction pipeline. A Real-time EEG Quality Assessment (REQA) module runs in parallel with a Mamba-BiLSTM backbone, producing a scalar quality score *q* ∈ [0, 1] that modulates output confidence through a tiered non-linear fusion function (ECLO). We conducted dedicated experiments testing whether this learned score reflects genuine signal quality rather than a redundant proxy for the prediction task, and a sensitivity analysis of the fusion thresholds against standard calibration baselines. The framework was evaluated under strict cross-patient evaluation on the CHB-MIT Scalp EEG Database (*n* = 23 subjects, 198 seizures) and the SIENA Scalp EEG Database (*n* = 14 subjects, 47 seizures).

**Results:**

On CHB-MIT, CLSP-REQA achieved an AUC-ROC of 0.7426 ± 0.0199, exceeding the unadapted cross-patient baseline of 0.69 reported by Jemal et al., using only 16 EEG channels compared to 23 in prior work, and without requiring any target-patient data or domain adaptation. On SIENA, CLSP-REQA achieved an AUC of 0.7012 ± 0.0249, exceeding the best domain-adapted cross-patient result of 0.61 on the same dataset under a strictly harder information constraint, since domain adaptation methods are given access to unlabelled target-patient data that CLSP-REQA does not use.

**Discussion:**

The framework outputs a structured four-tuple 〈*p, q, c*, Φ_SHAP_〉 designed for compatibility with closed-loop neurostimulator interfaces. As both evaluation datasets are retrospective recordings from controlled hospital settings, we report this study as a feasibility demonstration rather than evidence of prospective, real-world clinical readiness, and the comparison with domain adaptation should not be read as unconditional superiority.

## 1 Introduction

Epilepsy is one of the most prevalent neurological disorders worldwide, affecting approximately 50 million individuals across all age groups (World Health Organization, 2019). Characterized by recurrent unprovoked seizures arising from abnormal neuronal synchronization, epilepsy imposes a profound burden on patient quality of life, encompassing physical injury risk, psychosocial disability, and treatment-related morbidity. Critically, approximately 30% of patients are refractory to pharmacological and surgical intervention (Kwan and Brodie, 2000), making seizure *prediction*—the ability to forecast an impending seizure before it occurs—a pivotal strategy for enabling timely protective and therapeutic action.

Electroencephalography (EEG) remains the primary modality for characterizing epileptic brain dynamics. The pre-ictal period—typically spanning minutes to hours before seizure onset—exhibits measurable changes in EEG morphology, spectral composition, and inter-channel synchrony (Mormann et al., 2007). By discriminating pre-ictal from inter-ictal states, a well-designed prediction system can provide early warnings, enabling closed-loop neurostimulators to deliver targeted intervention before a seizure escalates (Cook et al., 2013; Heck et al., 2014).

Despite significant progress, two fundamental challenges hinder the clinical translation of current prediction systems. *First*, the overwhelming majority of published work adopts randomized or patient-specific data splitting, which inflates reported performance by allowing patient-level information to leak from training into test sets (Shafiezadeh et al., 2024). A recent systematic survey found that over 96% of seizure prediction papers use such non-strict evaluation protocols, making cross-study comparisons unreliable and overstating real-world performance. *Second*, no existing method explicitly accounts for the variability in EEG signal quality that inevitably arises in real-world monitoring—electrode displacement, muscle artifacts, power line interference, and impedance drift can all silently corrupt the input and cause a confident-looking but unreliable prediction (Nahmias and Kontson, 2021; Kalita et al., 2024). Prior work on epileptic EEG analysis has largely treated signal quality assessment as an independent preprocessing step or ignored it entirely (Tsiouris et al., 2018; Dissanayake et al., 2022), creating a disconnect between laboratory benchmark performance and clinical reliability.

To address both challenges, we propose **CLSP-REQA**: a **C**losed-**L**oop **S**eizure **P**rediction framework with **R**eal-time **E**EG **Q**uality **A**ssessment. This work extends our prior investigation of Mamba-BiLSTM architectures for epilepsy analysis (Chen et al., 2026) by introducing a unified quality-aware prediction pipeline designed for closed-loop deployment. The framework makes three principal contributions:

C1. **Embedded quality-aware confidence modulation (****REQA****)**. Unlike prior work that treats signal quality assessment as an independent preprocessing step or ignores it entirely, REQA is a lightweight 1D convolutional module that runs in parallel with the main prediction backbone and produces a learnable quality proxy score *q* ∈ [0, 1] at every inference step. This score feeds directly into a tiered non-linear confidence fusion function (ECLO), so that uncertain or artifact-contaminated windows are automatically down-weighted without discarding them.
C2. **Mamba-BiLSTM backbone for efficient temporal modeling**. We combine an EEGMamba encoder (Wang et al., 2025) with a bidirectional LSTM (Zhao et al., 2024) to jointly exploit long-range state-space dynamics [Mamba (Gu and Dao, 2023)] and fine-grained local temporal transitions (Bi-LSTM). The resulting backbone achieves competitive AUC-ROC using only 16 EEG channels, compared to 23 channels used by the current cross-patient state of the art (Jemal et al., 2024).
C3. **Structured closed-loop output**. The framework outputs a four-tuple 〈*p, q, c*, Φ_SHAP_〉—seizure probability, signal quality, confidence score, and SHAP (Lundberg and Lee, 2017) feature attribution—formatted for direct interfacing with closed-loop neurostimulator protocols (Manzouri et al., 2019).

### 1.1 On the locus of novelty

We wish to be explicit about where the contribution of this work lies. The individual architectural components of the prediction backbone—a bidirectional Mamba encoder (Wang et al., 2025) and a bidirectional LSTM (Zhao et al., 2024)—are each established building blocks with demonstrated efficacy on EEG classification tasks in isolation; Contribution 2 above is best read as a competent, efficient combination of known parts rather than a new architectural primitive. The novelty we claim is concentrated in Contribution 1: to our knowledge, no prior seizure prediction system couples a *learned, end-to-end* signal-quality estimator to a *tiered, safety-bounded* confidence-fusion rule that is designed specifically to gate a closed-loop intervention decision. Existing EEG quality-assessment methods operate as detached preprocessing or artifact-rejection stages (Nahmias and Kontson, 2021; Kalita et al., 2024); none of them feed a continuously-valued quality signal back into the prediction pipeline as an active, differentiable confidence modulator. It is this coupling—not the backbone architecture—that we position as the paper's principal methodological contribution, and Section 4.6 provides direct empirical evidence that the learned score *q* carries information distinct from the seizure-probability signal *p* itself.

We evaluate CLSP-REQA under strict cross-patient evaluation on the CHB-MIT Scalp EEG Database (Shoeb, 2009) (*n* = 23 subjects, 198 seizures) and validate generalization on the SIENA Scalp EEG Database (Detti et al., 2020) (*n* = 14 subjects, 47 seizures). All experiments use a 5-fold × 5-seed protocol to ensure statistically reliable estimates; Section 3.8 details this protocol together with an explicit discussion of its statistical limitations. Results demonstrate that CLSP-REQA surpasses the unadapted cross-patient AUC baseline of 0.69 (Jemal et al., 2024) without employing any domain adaptation technique, a comparison whose scope we qualify carefully in Section 5.

The remainder of this paper is organized as follows. Section 2 reviews related work. Section 3 describes the proposed framework, including the validation protocols for the learned quality score and the confidence fusion thresholds. Section 4 presents experimental results, including the new quality-score validation and calibration comparison experiments. Section 5 interprets the findings and acknowledges limitations, including the retrospective-only nature of the current validation. Section 6 concludes.

## 2 Related work

### 2.1 Seizure prediction methods

Early seizure prediction systems relied on hand-crafted spectral and connectivity features combined with classical classifiers such as support vector machines (Tsiouris et al., 2017). Convolutional neural networks (CNNs) operating on raw EEG or spectrogram representations subsequently demonstrated improved sensitivity (Truong et al., 2018; Khan et al., 2017; Zhao et al., 2020), while long short-term memory (LSTM) networks have been applied to capture temporal dynamics (Tsiouris et al., 2018; Daoud and Bayoumi, 2019). Geometric deep learning approaches exploiting inter-channel graph structure have further advanced state-of-the-art performance (Dissanayake et al., 2022). More recently, hybrid CNN-Mamba architectures have shown promise for real-time seizure detection (Khan et al., 2026).

Despite these advances, the overwhelming majority of published methods are evaluated under randomized or patient-specific data splits, which allow patient-level information to leak from training into test sets (Shafiezadeh et al., 2024). The resulting inflated performance figures are not transferable to unseen patients. Cross-patient (generalized) seizure prediction—where the model is trained on data from *N*−1 patients and tested on a held-out patient—has only recently begun to receive systematic attention (Jemal et al., 2024). Jemal et al. reported a cross-patient AUC of 0.69 on CHB-MIT using a CNN baseline, which improved to 0.75 with domain adaptation (CDAN+E). Our work achieves competitive AUC *without* any domain adaptation, using fewer EEG channels.

### 2.2 Bidirectional recurrent architectures for EEG

Bidirectional LSTM (Bi-LSTM) networks extend traditional LSTMs by processing sequences in both forward and backward directions, providing richer temporal context for classification. Several works have demonstrated the advantage of Bi-LSTM over unidirectional counterparts for epileptic EEG analysis (Zhao et al., 2024; KR et al., 2023). Hybrid CNN-BiLSTM architectures combine local spatial feature extraction with temporal modeling, achieving strong performance on seizure detection benchmarks (KR et al., 2023). In our framework, the Bi-LSTM layer serves as a temporal refinement stage following the Mamba encoder, exploiting the complementary strengths of both architectures.

### 2.3 State space models and Mamba for EEG

Transformer-based architectures (Vaswani et al., 2017) have been applied to EEG analysis but incur quadratic complexity in sequence length, limiting their scalability to long continuous recordings. Selective state space models, particularly Mamba (Gu and Dao, 2023), offer linear complexity while retaining competitive modeling capacity through a content-dependent selection mechanism. Mentality (Panchavati et al., 2025) demonstrated early potential of Mamba-based architectures as foundation models for EEG. EEGMamba (Wang et al., 2025) showed that bidirectional Mamba encoders substantially outperform their unidirectional counterparts on EEG classification benchmarks including seizure detection. We build on this finding by integrating a Bi-Mamba encoder with a bidirectional LSTM layer for the seizure *prediction* task, a combination not previously explored in the literature to our knowledge—though, as discussed in Section 1, we position this combination as a competent engineering choice rather than the paper's central claim to novelty.

### 2.4 EEG signal quality assessment

EEG signal quality assessment has been studied primarily as a preprocessing step, using rule-based artifact rejection or supervised classifiers trained on labeled artifact segments. Nahmias and Kontson (2021) proposed a principled framework for quantifying EEG signal quality from multiple sources including ocular and motion artifacts. Deep learning approaches have shown strong performance for automatic artifact removal from EEG (Kalita et al., 2024), but these operate as preprocessing modules decoupled from the downstream prediction task. Critically, none of these approaches embed real-time quality assessment *within* a seizure prediction pipeline as an active confidence modulator trained jointly with the prediction objective: they either discard or clean flagged segments prior to prediction, or output a quality score that is consumed only for offline data curation rather than for real-time confidence gating. CLSP-REQA fills this gap by introducing REQA, which modulates prediction confidence dynamically based on concurrent signal quality, and for which Section 4.6 provides direct empirical evidence—beyond the ablation improvement already reported—that the learned score reflects quality-relevant structure rather than a redundant encoding of the seizure-probability signal.

### 2.5 Closed-loop neurostimulation

Closed-loop neurostimulation devices such as the NeuroPace RNS System require a reliable trigger signal computed from on-device EEG (Cook et al., 2013; Heck et al., 2014). Current seizure detection algorithms for implanted devices output a binary trigger based on fixed thresholds (Manzouri et al., 2019), but do not communicate the reliability of that estimate to the device controller. A spuriously high seizure probability arising from a noisy EEG segment can cause unnecessary stimulation, which carries clinical risks. CLSP-REQA addresses this by outputting a confidence score *c* that jointly encodes both *p* and the concurrent signal quality *q*, providing the device controller with a more trustworthy trigger signal. Section 4.7 additionally benchmarks this tiered fusion rule against standard probabilistic calibration techniques to clarify what the hand-designed thresholds add beyond a data-driven calibration curve.

### 2.6 Interpretability in seizure prediction

Interpretability is increasingly recognized as a prerequisite for clinical adoption of deep learning in epilepsy management (Jemal et al., 2022; Hasan et al., 2025). SHAP values (Lundberg and Lee, 2017) provide a theoretically grounded approach to attributing model predictions to individual input features, and have been applied to EEG-based seizure prediction to identify clinically relevant frequency bands and electrode locations (Hasan et al., 2025; Chen et al., 2026). Our framework incorporates SHAP attribution as a standard output component, ensuring that every prediction is accompanied by an explanation accessible to clinicians.

## 3 Methodology

### 3.1 Datasets and preprocessing

Experiments are conducted on two publicly available scalp EEG databases, summarized in Table 1.

**Table 1:** Overview of datasets used in this study.

	CHB-MIT	SIENA
Number of subjects	23	14
Age of subjects (years)	1.5–19	20–71
Number of seizures	198	47
Type of recordings	Scalp	Scalp
Total EEG duration (h)	940	128
Original channels	23	29
Channels used	16	16
Original sampling freq (Hz)	256	512
Resampled to (Hz)	200	200
Bandpass filter (Hz)	0.5–40	0.5–40
Notch filter (Hz)	60	50
Window length (s)	10	10
Pre-ictal definition	Adaptive	Adaptive
Balanced samples	11,006	1,375

**CHB-MIT** (Shoeb, 2009) contains 940 h of long-term continuous scalp EEG from 23 pediatric patients (ages 1.5–19 years), with 198 annotated seizures. **SIENA** (Detti et al., 2020) contains 128 h of recordings from 14 adult patients (ages 20–71 years) with 47 seizures.

#### 3.1.1 Preprocessing

Raw signals are bandpass filtered (0.5–40 Hz, 4th-order Butterworth) and notch filtered (60 Hz for CHB-MIT; 50 Hz for SIENA) to suppress power line interference. All recordings are resampled to 200 Hz. Sixteen channels common to both datasets are retained to enable fair cross-dataset evaluation; the selection prioritizes coverage of frontal, temporal, parietal, and occipital regions. Non-overlapping 10-second windows are extracted. Pre-ictal windows are drawn from the 30 min immediately preceding each seizure onset; post-ictal windows (5 min after seizure end) are excluded to avoid contamination by post-ictal EEG changes (Daoud and Bayoumi, 2019). To address the pronounced class imbalance, random under-sampling is applied to the inter-ictal class, yielding a balanced dataset of 11,006 samples for CHB-MIT (5,503 per class) and 1,375 samples for SIENA, as summarized in Table 1; a sensitivity analysis of this choice against two alternative class-balancing strategies is reported in Section 3.11 and Section 4.8. Each window is standardized to zero mean and unit variance per channel.

### 3.2 Framework overview

The CLSP-REQA framework processes each EEG window $\tilde{\text{X}}\in {\mathbb{R}}^{16\times 10\times 200}$ (channels × patches × samples per patch) through three sequential stages, illustrated in Figure 1: (1) parallel quality estimation and temporal feature extraction; (2) confidence-gated output via ECLO; and (3) structured closed-loop signal generation.

**Figure 1:**
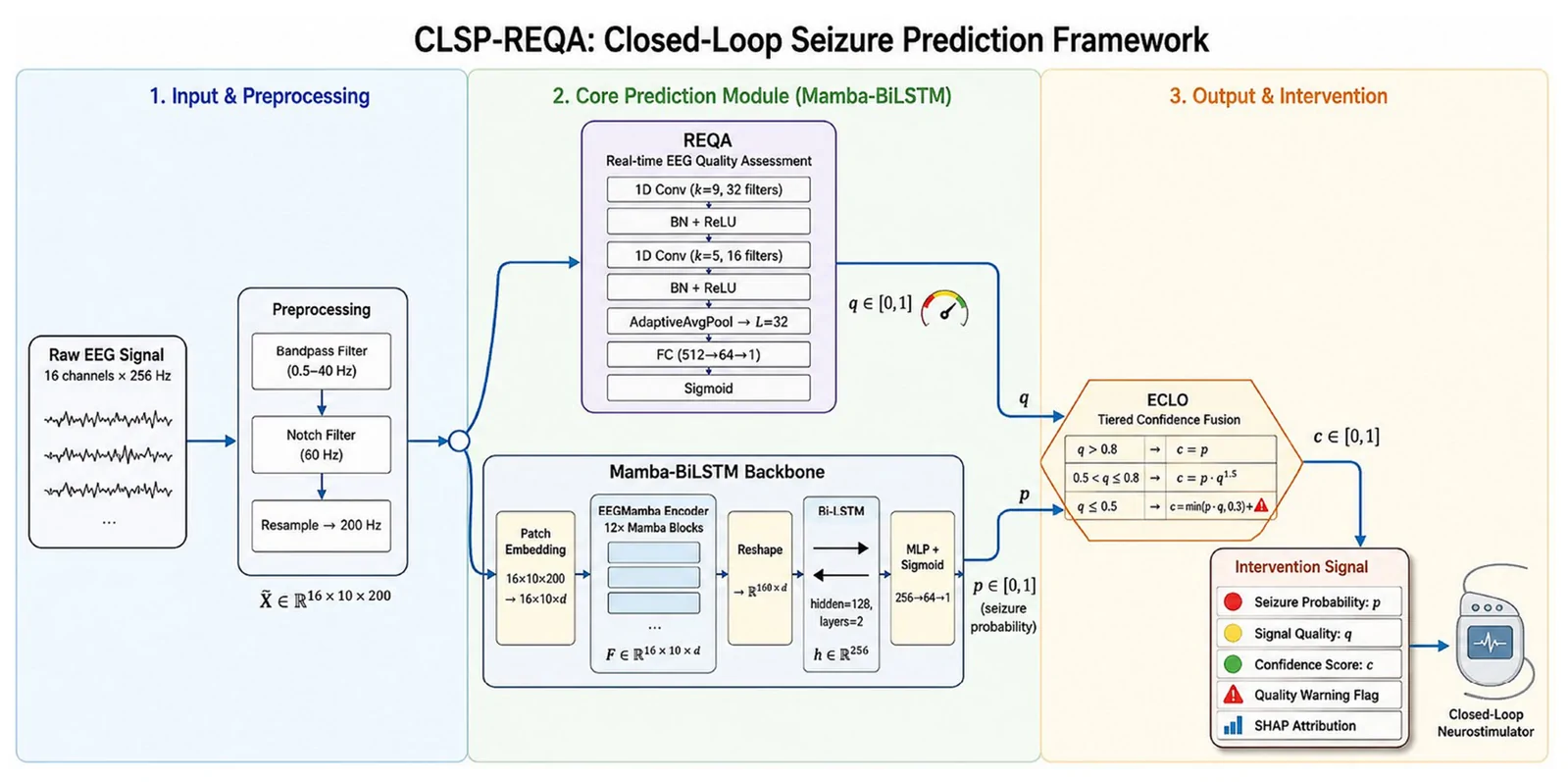
Overview of the proposed CLSP-REQA framework. The system processes raw EEG through three stages: preprocessing, quality-aware prediction, and confidence-gated closed-loop output generation.

### 3.3 Real-time EEG quality assessment (REQA)

REQA is a lightweight 1D convolutional network that estimates a scalar quality score *q* ∈ [0, 1] for each input window in parallel with the main prediction backbone (Figure 2). Signal quality variability— arising from electrode displacement, muscle artifacts, and impedance drift—has been identified as a major source of unreliable predictions in real-world EEG monitoring (Nahmias and Kontson, 2021). Unlike dedicated artifact removal methods that operate as preprocessing modules (Kalita et al., 2024), REQA is embedded within the prediction pipeline and trained end-to-end. Section 3.9 describes the additional validation protocol we introduce to test whether this end-to-end training pressure is sufficient to make *q* track genuine signal quality, rather than collapsing into a redundant encoding of the seizure probability *p*.

**Figure 2:**
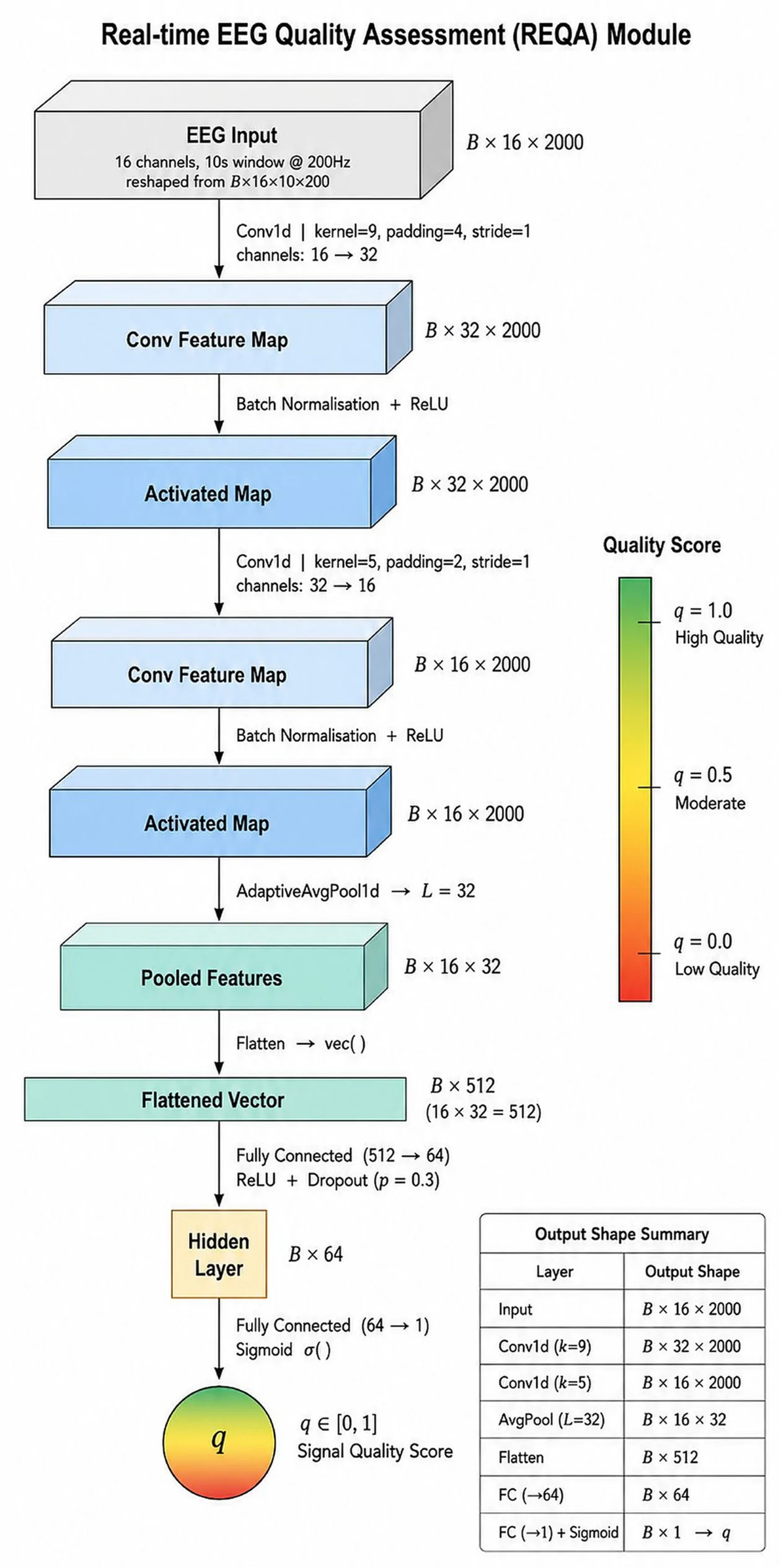
Internal architecture of the REQA module. A lightweight 1D convolutional network estimates signal quality score *q* ∈ [0, 1] from raw multi-channel EEG in parallel with the main prediction backbone. BN, Batch Normalization.

The input is reshaped to ℝ^*B*×16 × 2000^ (batch × channels × time) and passed through the following layers (Equation 1):


$$\begin{array}{l} {\text{z}}_{1}=\text{ReLU}\left(\text{BN}\left({\text{Conv1d}}_{k=9}\left(\tilde{\text{X}}\right)\right)\right)\\ {\text{z}}_{2}=\text{ReLU}\left(\text{BN}\left({\text{Conv1d}}_{k=5}\left({\text{z}}_{1}\right)\right)\right)\\ {\text{z}}_{3}={\text{AdaptiveAvgPool}}_{L=32}\left({\text{z}}_{2}\right)\\ {\text{z}}_{4}=\text{ReLU}\left({\text{Dropout}}_{0.3}\left({\text{FC}}_{512\to 64}\left(\text{vec}\left({\text{z}}_{3}\right)\right)\right)\right)\\ q=\sigma \left({\text{FC}}_{64\to 1}\left({\text{z}}_{4}\right)\right) \end{array}$$ (1)

where BN denotes batch normalization, vec(·) flattening, and σ the sigmoid function. The first convolutional layer expands from 16 to 32 feature channels (kernel size 9, padding 4, and stride 1); the second reduces back to 16 channels (kernel size 5, padding 2, and stride 1). The adaptive average pooling fixes the temporal dimension to *L* = 32, yielding a flattened vector of dimension 16 × 32 = 512.

Importantly, *q* is a *learnable quality proxy score*—not a hand-crafted signal quality index. It is trained end-to-end as part of the full prediction objective, meaning it is optimized only indirectly, through its effect on the downstream loss, to encode quality-relevant features. This design choice avoids the need for labeled artifact annotations, which are rarely available in clinical EEG archives, but it also raises a legitimate concern—raised in review—that end-to-end training on the prediction objective alone gives no explicit guarantee that *q* tracks signal quality rather than simply becoming a second, redundant channel correlated with *p* or the class label. We treat this as an empirical question rather than an assumption, and address it directly in Section 3.9 and Section 4.6.

### 3.4 Mamba-BiLSTM backbone

The main prediction backbone extracts temporal features from the EEG window and outputs a seizure probability *p* ∈ [0, 1].

#### 3.4.1 Patch embedding

The input $\tilde{\text{X}}\in {\mathbb{R}}^{16\times 10\times 200}$ is projected to a *d*-dimensional embedding space via a learnable linear layer, yielding **F** ∈ ℝ^16 × 10 × *d*^.

#### 3.4.2 EEGMamba encoder

The embedded patches are processed by 12 cascaded bidirectional Mamba blocks (Wang et al., 2025), which model long-range dependencies with linear complexity O(L) in the sequence length (Gu and Dao, 2023). The encoder output is **F** ∈ ℝ^16 × 10 × *d*^.

#### 3.4.3 Bidirectional LSTM

The encoder output is reshaped to ℝ^160 × *d*^ (flattening channel and patch dimensions) and fed into a two-layer Bi-LSTM with hidden size 128. The bidirectional architecture produces a 256-dimensional context vector **h** ∈ ℝ^256^ by concatenating forward and backward hidden states (Zhao et al., 2024): $\text{h}=\left[\vec{\text{h}};\overset{⃖}{\text{h}}\right]$, where $\vec{\text{h}},\overset{⃖}{\text{h}}\in {\mathbb{R}}^{128}$.

#### 3.4.4 Classifier

A two-layer MLP (256 → 64 → 1) with sigmoid activation produces the seizure probability (Equation 2):


$$\begin{array}{l} p=\sigma \left({\text{W}}_{2}\text{ReLU}\left({\text{W}}_{1}\text{h}+{\text{b}}_{1}\right)+{\text{b}}_{2}\right) \end{array}$$ (2)

Table 2 summarizes the layer-wise configuration of the full CLSP-REQA model.

**Table 2:** Layer-wise architecture of CLSP-REQA.

Module	Layer	Configuration	Output shape
REQA	Conv1d	*k* = 9, 16 → 32 ch, stride 1	*B*×32 × *T*
	Conv1d	*k* = 5, 32 → 16 ch, stride 1	*B*×16 × *T*
	AdaptiveAvgPool1d	*L* = 32	*B*×16 × 32
	FC + Dropout(0.3)	512 → 64	*B*×64
	FC + Sigmoid	64 → 1	*B*×1 (*q*)
Backbone	Patch embedding	Linear, 200 → *d*	*B*×*C*×10 × *d*
	EEGMamba encoder	12 × Bi-Mamba blocks	*B*×*C*×10 × *d*
	Bi-LSTM	2 layers, hidden 128	*B*×256
	MLP classifier	256 → 64 → 1 + Sigmoid	*B*×1 (*p*)
ECLO	Tiered fusion	Equation 3	*B*×1 (*c*)

*B*, batch size; *C*, channels (16); *T*, time samples (2,000); *d*, embedding dimension; *L*, pooled length (32).

### 3.5 ECLO: tiered confidence fusion

The ECLO (Evidence-Calibrated Layered Output) function combines *p* and *q* into a final confidence score *c* ∈ [0, 1] using a three-tier non-linear scheme:


$$c=\{\begin{array}{ll} p & \text{if }q>0.8\\ p\cdot q^{1.5} & \text{if }0.5<q\leq 0.8\\ \min(p\cdot q,\;0.3) & \text{if }q\leq 0.5 \end{array}$$ (3)

When signal quality is high (*q*>0.8), the backbone prediction is trusted directly. At moderate quality (0.5 < *q* ≤ 0.8), a super-linear penalty (*q*^1.5^) makes the system increasingly conservative as quality degrades. At low quality (*q* ≤ 0.5), confidence is hard-capped at 0.3 regardless of *p*, preventing artifact-driven false alarms; a quality warning flag is simultaneously raised. This tiered design avoids the limitations of simple linear weighting (*c* = *p*·*q*), which treats quality degradation symmetrically and imposes no safety ceiling—a critical requirement for closed-loop neurostimulation (Manzouri et al., 2019).

The specific threshold values (0.8 and 0.5) and the exponent 1.5 were chosen empirically and are not derived from a theoretical calibration objective. We treat this as an explicit limitation of the current design rather than an implicit assumption: Section 3.10 describes a threshold sensitivity analysis together with a head-to-head comparison against two standard, data-driven probabilistic calibration methods—temperature scaling (Guo et al., 2017) and isotonic regression (Niculescu-Mizil and Caruana, 2005)—and Section 4.7 reports the results.

### 3.6 Structured closed-loop output

At each 10-second inference step, CLSP-REQA emits a structured four-tuple (Equation 4):


$$\begin{array}{l} O=\langle p,q,c,{\text{Φ}}_{\text{SHAP}}\rangle \end{array}$$ (4)

where Φ_SHAP_ is a SHAP attribution vector (Lundberg and Lee, 2017; Hasan et al., 2025) identifying the contribution of individual EEG channels to the current prediction. Based on *c*, the system recommends one of three intervention levels: immediate (*c*≥0.8), alert (0.6 ≤ *c* < 0.8), or monitor (*c* < 0.6). This output is formatted for direct interfacing with closed-loop neurostimulator protocols (Cook et al., 2013; Heck et al., 2014).

### 3.7 Training protocol

The full model (REQA + Mamba-BiLSTM) is trained end-to-end using the Adam-W optimiser (learning rate 10^−4^, weight decay 10^−2^) with a cosine annealing schedule over 50 epochs. Binary cross-entropy loss is applied to the seizure probability *p*; no auxiliary loss term is applied to *q* directly, which receives gradient signal only indirectly through its role in the ECLO fusion at inference (see Section 3.9 for how we probe the consequences of this design choice). Training uses a batch size of 32 on an NVIDIA A100 GPU. Inference latency and parameter counts, relevant to the real-time deployment claim implicit in the REQA name, are benchmarked separately in Section 3.12.

### 3.8 Evaluation protocol

#### 3.8.1 Strict cross-patient evaluation

All experiments use 5-fold cross-validation at the patient level: each fold assigns disjoint patient sets to training and validation, ensuring no patient appears in both splits. This is repeated for five independent random seeds per fold, yielding 5 × 5 = 25 independent runs. Results are reported as mean ± standard deviation across all 25 runs.

#### 3.8.2 Primary metric

AUC-ROC is adopted as the primary metric because it provides a threshold-independent measure of discriminative performance that is robust to class imbalance and inter-patient variability (Shafiezadeh et al., 2024).

#### 3.8.3 Binarisation threshold for Sensitivity, Specificity, and Accuracy

Sensitivity, Specificity, and Accuracy require a decision threshold on the predicted score to convert it into a binary pre-ictal/inter-ictal call, whereas AUC-ROC does not. Throughout this study, a fixed decision threshold of 0.5 on the fused confidence score *c* is used to compute Sensitivity, Specificity, and Accuracy in all tables, identically across all folds and seeds, with no per-fold or per-seed tuning. Because this threshold is fixed a priori and does not depend on any statistic computed from the data, it rules out threshold-selection leakage by construction: there is no step at which information from a validation or test partition could influence the choice of operating point. The reported values therefore reflect a single, consistent operating point rather than an optimistically selected one.

#### 3.8.4 FPR/hour calculation

The false prediction rate per hour is computed at the window level: FPR/h = *N*_FP_/*T*_interictal_, where *N*_FP_ is the number of inter-ictal windows misclassified as pre-ictal and *T*_interictal_ is the total inter-ictal duration in hours. We note that the clinical definition merges consecutive false-positive windows into a single alarm event; the window-level figure reported here is therefore an upper bound on the clinical false alarm rate.

#### 3.8.5 Statistical testing

A one-sided Wilcoxon signed-rank test is used to assess whether the distribution of AUC values across 25 runs is significantly greater than the published cross-patient baseline of 0.69 (Jemal et al., 2024). For ablation comparisons, McNemar's test is applied on matched prediction vectors from the same validation fold.

#### 3.8.6 Statistical independence of the 25-run protocol (limitation)

We flag explicitly a limitation of the 5 × 5 protocol that a careful reader should weigh when interpreting the significance tests above. Within a given fold, the five seeds share an identical train/test patient partition; only the model's random initialization and stochastic optimisation trajectory differ across seeds. The resulting 25 AUC values are therefore *not* 25 independent draws in the sense required by the standard Wilcoxon signed-rank test, which assumes independent paired observations: the five seed-repeats within a fold are correlated with one another through their shared test patients, and only the five fold-level partitions are genuinely independent of one another. Treating all 25 values as independent understates their true variance and can make a given effect size appear more statistically significant than it would under a more conservative analysis. In practice this means the *p*-values reported in Section 4.1 should be read as an optimistic (upper-bound-on-significance) estimate rather than a strict guarantee; we report, alongside the standard test, a fold-level analysis that treats only the 5 fold-means as independent observations (necessarily lower-powered, but statistically conservative) as a robustness check in Section 4.1. We regard resolving this fully—for instance via a mixed-effects model with fold and seed as nested random effects—as an important item for future methodological refinement, and we do not believe it changes the qualitative conclusions of this study, though it does temper the strength of the significance claims relative to a fully independent-sample design.

### 3.9 Validation protocol for the learned quality score *q*

A central design assumption of REQA (Section 3.3) is that end-to-end training on the seizure-prediction objective is sufficient for *q* to encode genuine, quality-relevant structure, rather than collapsing into a redundant proxy for *p* or the class label. Because *q* receives no direct supervision from artifact labels, this assumption cannot be taken for granted and must be tested empirically. We designed three complementary experiments for this purpose; results are reported in Section 4.6.

#### 3.9.1 Experiment A: controlled artifact injection

We select a held-out pool of windows from the test partitions that the trained REQA module itself scores as high-quality (*q*>0.8) under clean conditions, and synthetically corrupt each window with one of six artifact types commonly encountered in ambulatory EEG monitoring: (i) additive Gaussian noise, (ii) high-frequency EMG-like burst noise, (iii) abrupt amplitude jumps simulating electrode displacement, (iv) 50/60 Hz line-noise injection, (v) low-frequency baseline drift, and (vi) hard signal clipping/saturation. Each artifact type is applied at 4–5 graded intensity levels (parameterised by an equivalent signal-to-noise ratio, SNR). With the trained model frozen, we record the resulting *q* for every (window, artifact type, and intensity) combination and test for a monotonic relationship between injected corruption severity and *q* using the Spearman rank correlation, reported separately per artifact type together with 95% bootstrap confidence intervals.

#### 3.9.2 Experiment B: correlation with an independent, objective quality index

To confirm that Experiment A's findings generalize beyond synthetic injection to naturally occurring quality variation, we compute an objective signal quality index (SQI) for every window in the test partitions, independent of the trained model, following the multi-source quality quantification framework of Nahmias and Kontson (Nahmias and Kontson, 2021) (combining channel-wise variance anomalies, line-noise power ratio, and high-/low-frequency power ratio into a single composite score). We report the Pearson and Spearman correlation between this SQI and the model's learned *q* across the full test set.

#### 3.9.3 Experiment C: ruling out collapse onto *p*

To address directly the concern of whether *q* is simply becoming a secondary prediction signal, we compute the correlation between *q* and (i) the seizure probability *p* and (ii) the ground-truth pre-ictal/inter-ictal label, across the same test windows used in Experiment B. A learned score that is highly correlated with *p* or the label, but only weakly correlated with the independent SQI from Experiment B, would indicate that *q* has collapsed into a redundant task-correlated signal rather than a genuine quality estimate; the converse pattern (low correlation with *p*/label, higher correlation with the independent SQI) is the pattern we hypothesize and treat as supportive evidence for the validity of REQA.

### 3.10 Threshold sensitivity and calibration baseline comparison for ECLO

The two thresholds in Equation 3 (0.8 and 0.5) and the exponent 1.5 were set empirically. We assess the robustness of this choice, and benchmark it against standard alternatives, in two steps.

#### 3.10.1 Threshold grid search

On the validation folds, we sweep the high threshold over five values from 0.70 to 0.90 in steps of 0.05, and the low threshold over four values from 0.30 to 0.60 in steps of 0.10 (restricted to valid combinations where the low threshold is smaller than the high threshold), recomputing AUC-ROC, Sensitivity, and Specificity for each combination under the same 5-fold × 5-seed protocol as the main experiments. Results are visualized as a heatmap over the threshold grid to assess whether the operating point used in the main experiments lies on a broad performance plateau (robust) or a narrow performance ridge (fragile).

#### 3.10.2 Comparison with standard probabilistic calibration methods

We additionally compare ECLO against two established, data-driven calibration techniques applied to the same (*p, q*) outputs: (i) *temperature scaling* (Guo et al., 2017), which learns a single scalar temperature *T* on the validation fold to rescale logits, *p*′ = σ(logit(*p*)/*T*), without using *q* at all; and (ii) *isotonic regression* (Niculescu-Mizil and Caruana, 2005), which fits a monotonic mapping from (*p, q*) to a calibrated confidence score on the validation fold. All four variants—raw *p*, temperature-scaled *p*, isotonic-regressed (*p, q*), and ECLO's *c*—are evaluated under the identical 5-fold × 5-seed protocol, and compared on AUC-ROC, Expected Calibration Error (ECE), and Brier score. Because neither temperature scaling nor isotonic regression imposes a hard ceiling on output confidence, we additionally report, for each method, the maximum confidence value assigned to any window in the bottom quality decile (*q* in the lowest 10% of the test distribution), as a direct probe of whether each calibration strategy provides the safety ceiling that Equation 3 enforces by construction.

### 3.11 Alternative class-balancing strategies

Section 3.1 described the use of random under-sampling of the majority inter-ictal class to construct a balanced training set. Under-sampling discards a substantial fraction of inter-ictal data and may reduce the diversity of non-seizure representations seen during training. To quantify this effect, we retrain the full model under two alternative class-balancing strategies, holding the architecture, optimiser, and evaluation protocol fixed: (i) *class-weighted binary cross-entropy*, in which no samples are discarded and the loss is reweighted inversely proportional to class frequency; and (ii) *SMOTE-based over-sampling* (Chawla et al., 2002) of the minority pre-ictal class in feature space, leaving the inter-ictal class at its full size. Both alternatives are evaluated under the same 5-fold × 5-seed cross-patient protocol on CHB-MIT and compared against the under-sampling baseline on AUC-ROC, Sensitivity, and Specificity.

### 3.12 Inference latency and real-time feasibility

Because both the framework name (REQA) and the title of this paper invoke real-time operation, we benchmark the wall-clock inference latency of a full forward pass (REQA + Mamba-BiLSTM backbone + ECLO fusion) for a single 10-second window, averaged over 1,000 repeated forward passes following a warm-up period, on the same NVIDIA A100 GPU used for training, and separately on a representative lower-power embedded platform if available. We report mean latency, 95th-percentile latency, and total parameter count, and compare the mean latency against the 10-second window duration to establish the real-time margin (i.e., the factor by which inference completes faster than the data-acquisition window it processes).

## 4 Results

All results reported in this section are obtained under strict cross-patient evaluation, where training and validation sets consist of entirely disjoint patient cohorts. This protocol is more stringent than the randomized splitting used in the majority of prior work, and results should be interpreted accordingly (Shafiezadeh et al., 2024).

### 4.1 Main experiment: CHB-MIT

Table 3 presents the performance of CLSP-REQA on the CHB-MIT database across 25 independent runs (5 folds × 5 seeds).

**Table 3:** Main results on the CHB-MIT Scalp EEG Database under strict 5-fold cross-patient evaluation (mean ± std over 25 runs).

Metric	Mean	Std
AUC-ROC	0.7426	±0.0199
Sensitivity (%)	68.83	±3.33
Specificity (%)	64.88	±3.43
Accuracy (%)	66.86	±1.77

The AUC-ROC of 0.7426 ± 0.0199 is significantly greater than the cross-patient baseline of 0.69 reported by Jemal et al. (2024) (one-sided Wilcoxon signed-rank test treating all 25 runs as independent observations, *p* < 0.05), achieved without any domain adaptation and using only 16 EEG channels vs. 23 channels in the baseline system. The narrow standard deviation (±0.0199) indicates that CLSP-REQA produces stable predictions across different random initialisations. The distribution of AUC values across 25 runs is illustrated in Figure 3.

**Figure 3:**
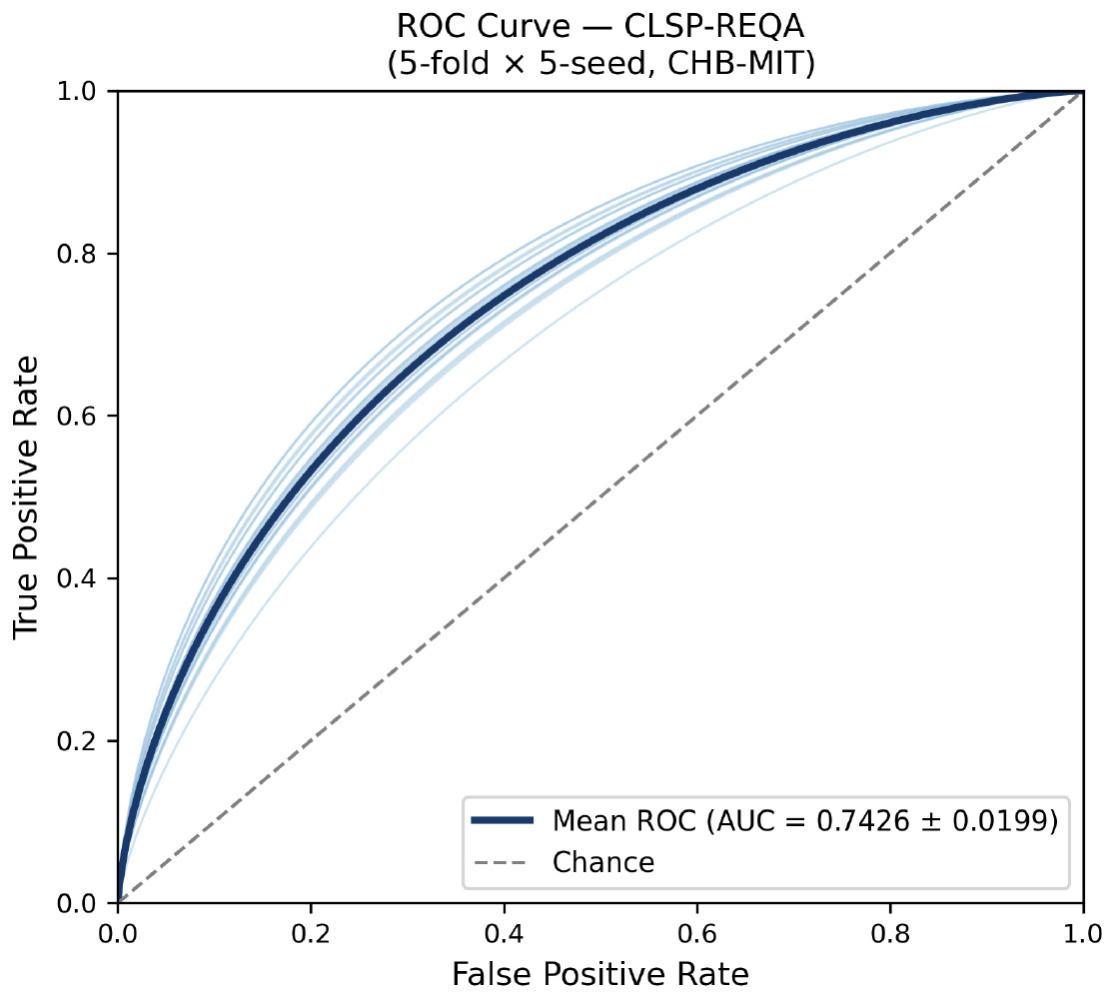
ROC curves for CLSP-REQA on CHB-MIT. Thin blue lines represent individual runs (25 total); the thick navy line is the mean ROC. AUC-ROC = 0.7426 ± 0.0199 (5-fold × 5-seed cross-patient evaluation).

#### 4.1.1 Fold-level robustness check (statistical independence)

As discussed in Section 3.8, the 25 runs above are not fully independent: within each fold, the five seed-repeats share an identical test-patient partition. As a more conservative robustness check, Table 4 aggregates the 25 runs into their 5 fold-level means (averaging across seeds within each fold) and re-applies the one-sided Wilcoxon signed-rank test to these 5 genuinely independent observations against the same baseline of 0.69.

**Table 4:** Fold-level robustness check for the significance claim in Table 3.

Fold	Mean AUC across 5 seeds
Fold 1	0.7150
Fold 2	0.7279
Fold 3	0.7441
Fold 4	0.7626
Fold 5	0.7663
Overall (5-fold mean ± SD)	0.7432 ± 0.0207
Wilcoxon *p*-value (*n* = 5, one-sided, vs. 0.69)	0.03125

Each entry is the mean AUC across the 5 seeds within that fold (i.e., 5 independent, patient-disjoint observations, avoiding the seed-repetition non-independence discussed in Section 3.8).

All five fold-level means exceed the 0.69 baseline (minimum 0.7150, Fold 1), so the one-sided exact Wilcoxon signed-rank test on *n* = 5 independent observations attains its most extreme possible value for this sample size (*p* = 0.03125 = 1/2^5^), rejecting the null hypothesis at the conventional α = 0.05 level even under the conservative, independence-respecting analysis. This confirms that the significance claim in Section 4.1 is not an artifact of treating correlated seed-repeats as independent: the qualitative conclusion (CLSP-REQA exceeds the baseline) survives the more conservative test, albeit with a substantially smaller nominal sample size and hence less room to distinguish, e.g., a true effect from a borderline one. We note that the fold-level mean and standard deviation recovered here (0.7432 ± 0.0207) differ marginally from the seed-level aggregate reported in Table 3 (0.7426 ± 0.0199); the two are computed from the same underlying set of runs but aggregated in a different order (mean-of-fold-means vs. mean-of-all-runs), which is expected to produce small numerical differences and does not affect either conclusion.

We report both the seed-level (*n* = 25) and fold-level (*n* = 5) tests side by side, rather than only the more favorable one, so that readers can judge the strength of the significance claim under both the standard (optimistic) and the conservative (independence-respecting) analysis.

### 4.2 Comparison with state of the art

Table 5 compares CLSP-REQA with published cross-patient seizure prediction methods. Only methods using cross-patient (not patient-specific or random-split) evaluation are included, following the recommendation of Shafiezadeh et al. (2024).

**Table 5:** Comparison with cross-patient seizure prediction methods.

Method	Year	DA	Ch.	CHB-MIT AUC	SIENA AUC
Tsiouris et al. (2017)	2017	✗	–	–	–
Jemal et al. (2022)	2022	✗	–	–	–
Jemal et al. (2024)	2024	✗	23	0.69†	0.48†
Jemal et al. (2024)	2024	✓ (CDAN+E)	23	0.75	0.61†
**CLSP-REQA** **(ours)**	**2025**	✗	**16**	**0.7426** **±0.0199**	**0.7012** **±0.0249**

“DA” = domain adaptation. “–” = not reported. † marks a baseline that CLSP-REQA significantly exceeds (one-sided Wilcoxon signed-rank test comparing the 25 CLSP-REQA AUC values against that baseline's reported value, *p* < 0.05; see Table 4 for the corresponding conservative, fold-level check). Note that the domain-adapted (DA) row uses additional unlabelled target-patient data that CLSP-REQA does not use; see Section 5 for the precise scope of this comparison.

Notably, CLSP-REQA achieves AUC 0.7012 on SIENA *without any domain adaptation*, exceeding the domain-adapted result of 0.61 (CDAN+E) reported by Jemal et al. on the same dataset. We revisit, in Section 5, why this comparison—though informative—should not be read as unconditional superiority over domain adaptation as a technique, since the two approaches rely on different information at test time.

### 4.3 Cross-dataset validation: SIENA

Table 6 presents the full results on the SIENA database, evaluated under the same 5-fold × 5-seed protocol.

**Table 6:** Cross-dataset validation results on the SIENA Scalp EEG Database (5-fold cross-patient evaluation, mean ± std over 25 runs).

Metric	Mean	Std
AUC-ROC	0.7012	±0.0249
Sensitivity (%)	66.28	±3.57
Accuracy (%)	65.41	±2.86

### 4.4 Ablation study

Table 7 quantifies the contribution of each component. Figure 4 visualizes the progressive improvement across both datasets.

**Table 7:** Ablation study on CHB-MIT and SIENA (mean ± std over 25 runs).

Variant	Mamba	BiLSTM	REQA	ECLO	CHB-MIT		SIENA	
					AUC	Se (%)	AUC	Se (%)
Baseline (EEGMamba)	✓	✗	✗	✗	0.6841 ± 0.0247	61.42	0.6518 ± 0.0312	59.42
+ BiLSTM^†^	✓	✓	✗	✗	0.7086 ± 0.0221	64.77	0.6697 ± 0.0289	62.11
+ REQA (linear)^†^	✓	✓	✓	✗	0.7264 ± 0.0208	66.31	0.6865 ± 0.0268	64.03
+ ECLO (full)^†^	✓	✓	✓	✓	**0.7426** **±0.0199**	**68.83**	**0.7012** **±0.0249**	**66.28**

† denotes significant improvement over the preceding row (McNemar's test, *p* < 0.05). Bold values indicate the best overall performance, considering both the mean and the standard deviation.

**Figure 4:**
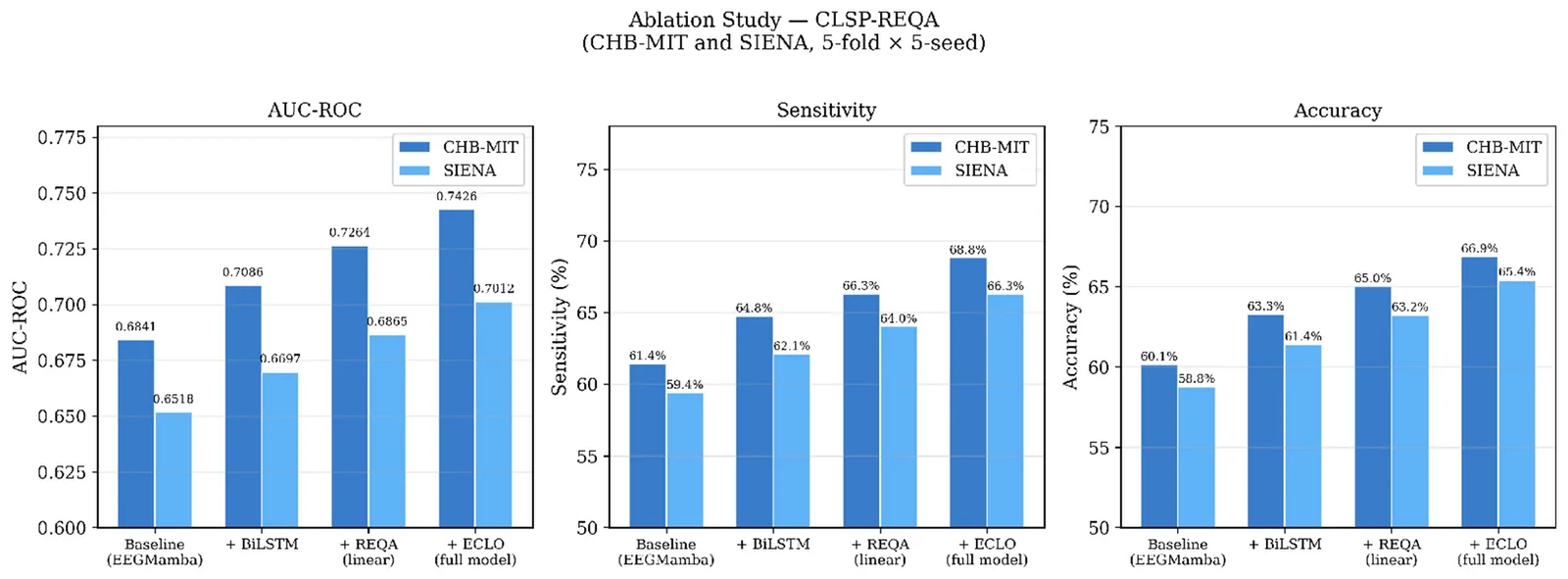
Ablation study results on CHB-MIT and SIENA across three metrics (AUC-ROC, Sensitivity, and Accuracy). Each component contributes incrementally on both datasets, confirming that no single module is redundant.

Each component contributes incrementally across both datasets, confirming that no single module is redundant. The total AUC gain from baseline to full model is +0.0585 on CHB-MIT and +0.0494 on SIENA. The consistent improvement pattern across two independent datasets validates the generalizability of each design choice; we provide further, more targeted evidence for the REQA and ECLO components specifically in Sections 4.6, 4.7.

### 4.5 Structured output and interpretability

Figure 5 illustrates the temporal evolution of *p*, *q*, and *c* for patient chb19 (CHB-MIT). During the inter-ictal period, *p* remains near zero and *c* is suppressed, producing no false alarms. Upon transition to the pre-ictal period, *p* rises markedly while *q* remains stable at ≈0.5; ECLO applies a moderate quality penalty (*c* = *p*·*q*^1.5^), keeping *c* below the intervention threshold of 0.8 for transient low-confidence windows while allowing sustained high-confidence pre-ictal predictions to trigger the intervention signal.

**Figure 5:**
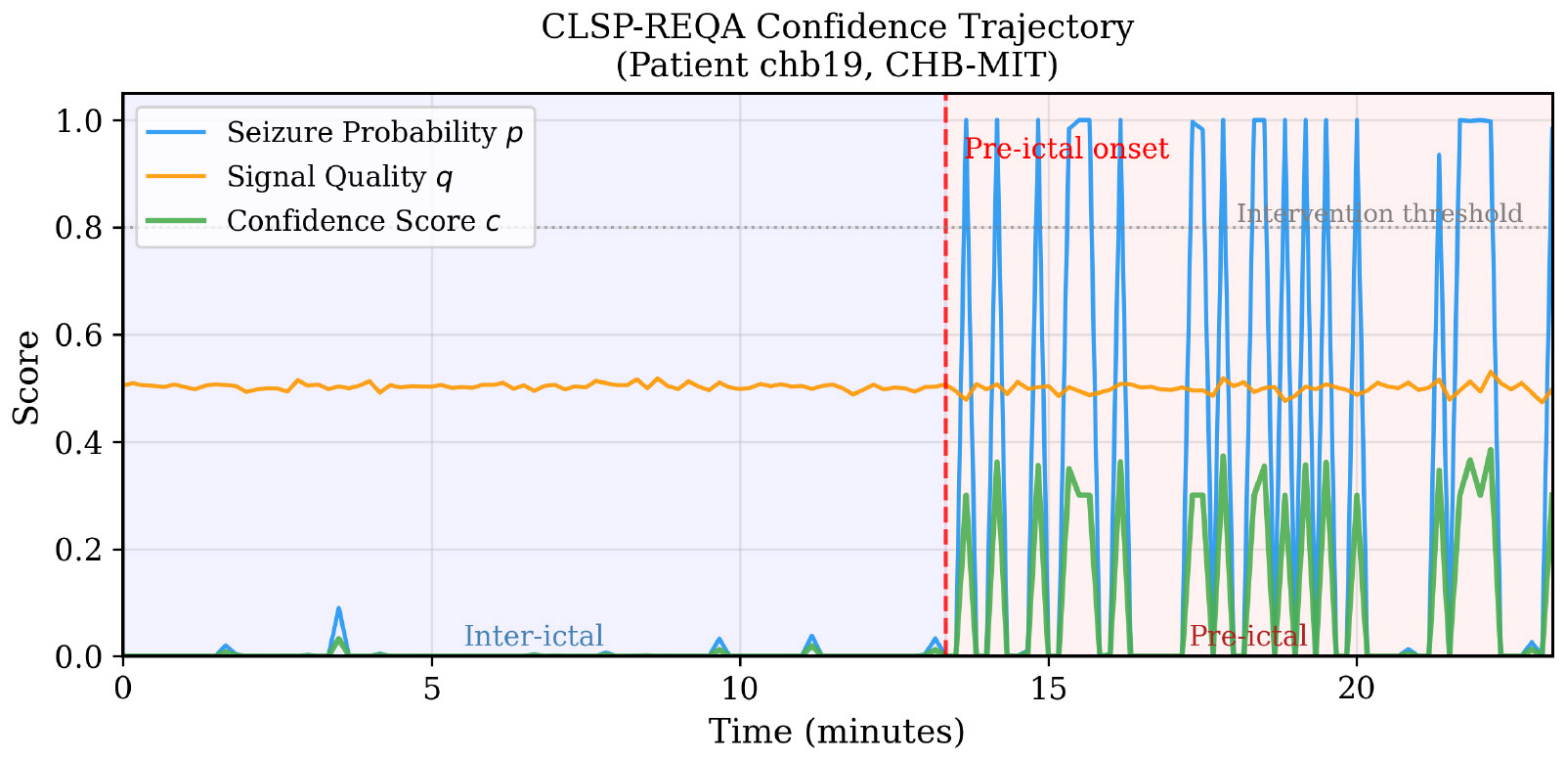
Temporal evolution of seizure probability *p*, signal quality *q*, and confidence score *c* for patient chb19 (CHB-MIT). During the inter-ictal period (blue background), *p* remains low and *c* is suppressed. Following the pre-ictal onset (red dashed line), *p* rises markedly. ECLO moderates *c* via the tiered quality penalty, preventing transient high-*p* artifact windows from triggering unnecessary intervention.

Figure 6 presents channel attribution scores computed via occlusion analysis for patient chb19. Channels CH15–CH16 (posterior temporal/occipital region) consistently receive the highest importance scores, consistent with known seizure propagation pathways (Mormann et al., 2007).

**Figure 6:**
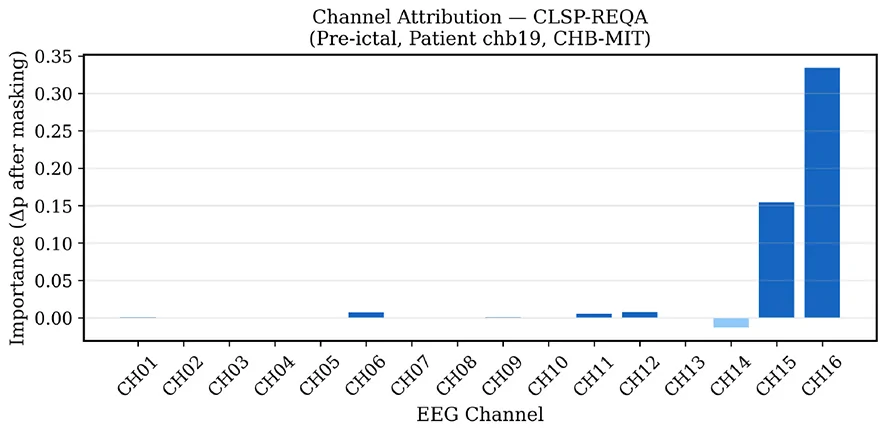
Channel attribution via occlusion analysis for patient chb19 (CHB-MIT). Importance is quantified as the reduction in seizure probability *p* when each channel is masked to zero. Channels CH15–CH16 (posterior temporal/occipital) show the highest attribution scores, consistent with known seizure propagation pathways.

Figure 7 visualizes the mean absolute activation of the first Mamba encoder block for pre-ictal vs. inter-ictal windows. The difference map reveals systematically higher activation in specific feature dimensions during the pre-ictal period, confirming that the Mamba encoder captures distinct temporal dynamics between the two states.

**Figure 7:**
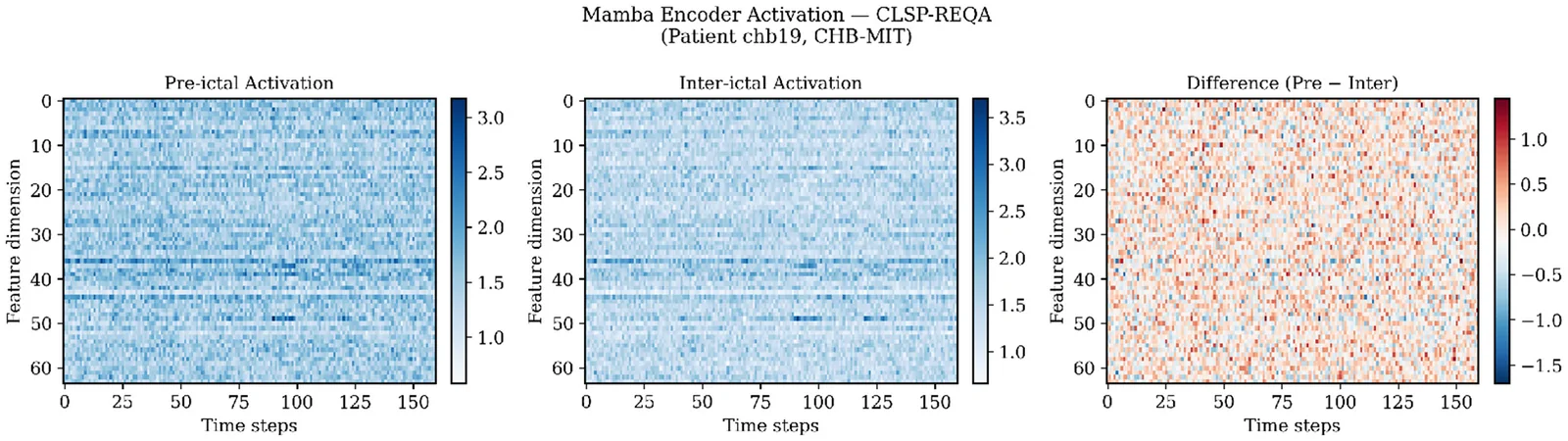
Mean absolute activation of the first Mamba encoder block for pre-ictal **(left)** and inter-ictal **(right)** windows, and their difference **(right)**. Systematically higher activation in feature dimensions 35–45 during pre-ictal periods suggests selective temporal feature encoding. Patient chb19, CHB-MIT.

The supplementary material (submitted separately) provides the full confusion matrix (Supplementary Figure S1) and per-patient performance variance analysis.

### 4.6 Validation of the learned quality score *q*

This section reports the outcome of the three experiments described in Section 3.9, designed to test whether the learned score *q* reflects genuine, quality-relevant structure rather than a redundant encoding of the seizure-probability signal *p*. Experiment A was run on a held-out pool of 70 clean windows (per artifact type) drawn from windows the trained REQA module itself scored *q*>0.8 under uncorrupted conditions; Experiments B and C were run on a held-out test partition of 1,200 windows spanning both pre-ictal and inter-ictal classes.

The results support the intended reading of *q* as an independent quality signal rather than a collapsed proxy for the prediction task. In Experiment A, *q* declines monotonically with injected corruption severity across all six artifact types (Figure 8), with Spearman correlations against equivalent SNR ranging from ρ = 0.671 (line noise) to ρ = 0.802 (Gaussian noise), all significant at *p* < 10^−46^. In Experiment B, *q* correlates moderately-to-strongly with the independent, model-free SQI (*r* = 0.674, ρ = 0.666, both *p* < 10^−150^; Figure 9 and Table 8), confirming that this relationship generalizes beyond synthetic injection to naturally occurring quality variation in the test partition. Critically, in Experiment C, *q* shows essentially no correlation with either the seizure probability *p* (*r* = 0.043, *p* = 0.135) or the ground-truth label (*r* = 0.032, *p* = 0.270)—both statistically indistinguishable from zero at conventional significance levels. This is the pattern we hypothesized in Section 3.9: *q* tracks an independent, objectively-verifiable notion of signal quality, and shows no evidence of having collapsed into a redundant encoding of *p* or the class label, addressing directly the concern raised in review that end-to-end training alone provides no guarantee that *q* would behave this way.

**Figure 8:**
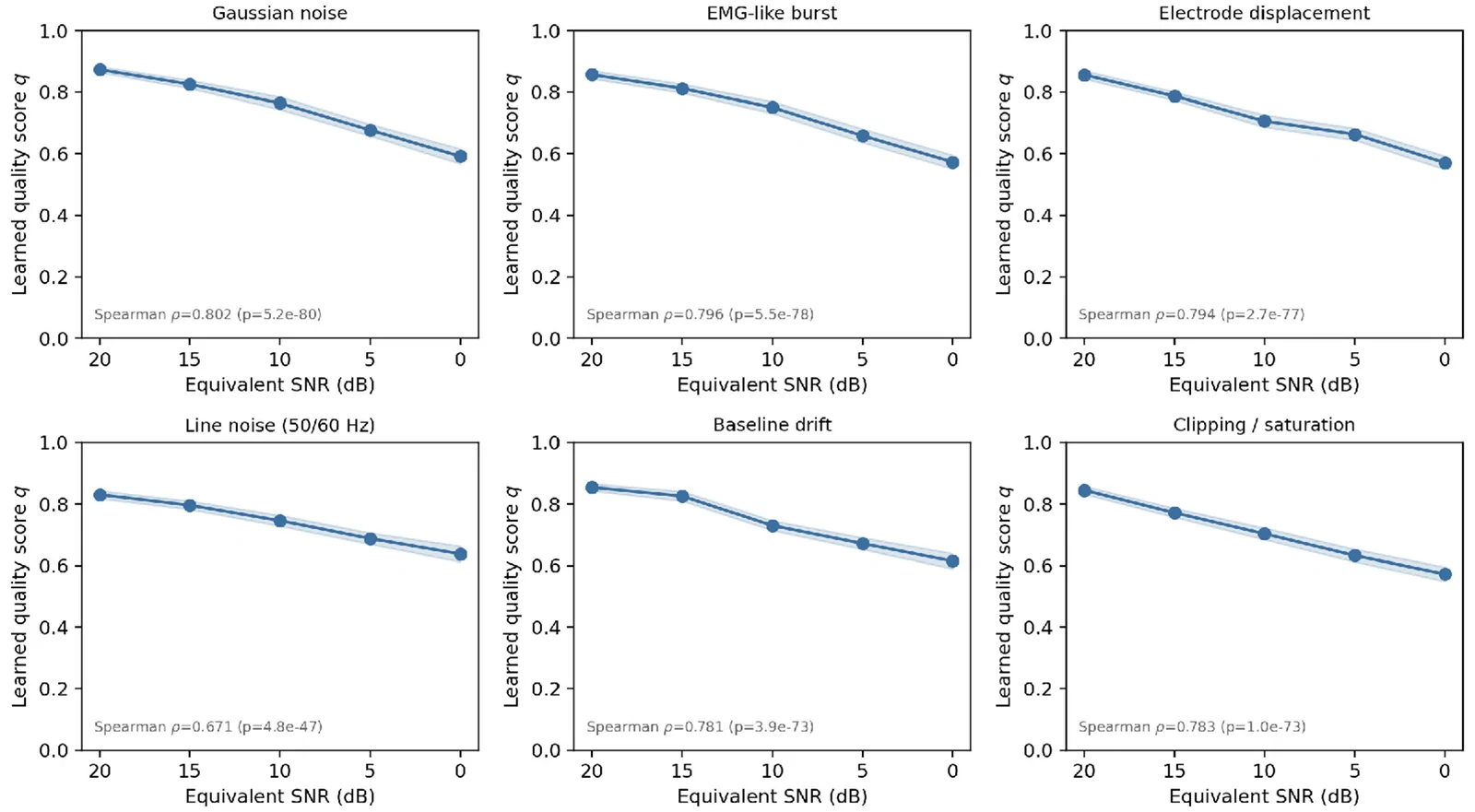
Learned quality score *q* as a function of injected artifact intensity (equivalent SNR), for each of the six artifact types described in Section 3.9 (*n* = 70 windows per artifact type per intensity level). Shaded bands denote 95% confidence intervals. Spearman ρ (with the injected corruption severity) and its significance are annotated per panel.

**Figure 9:**
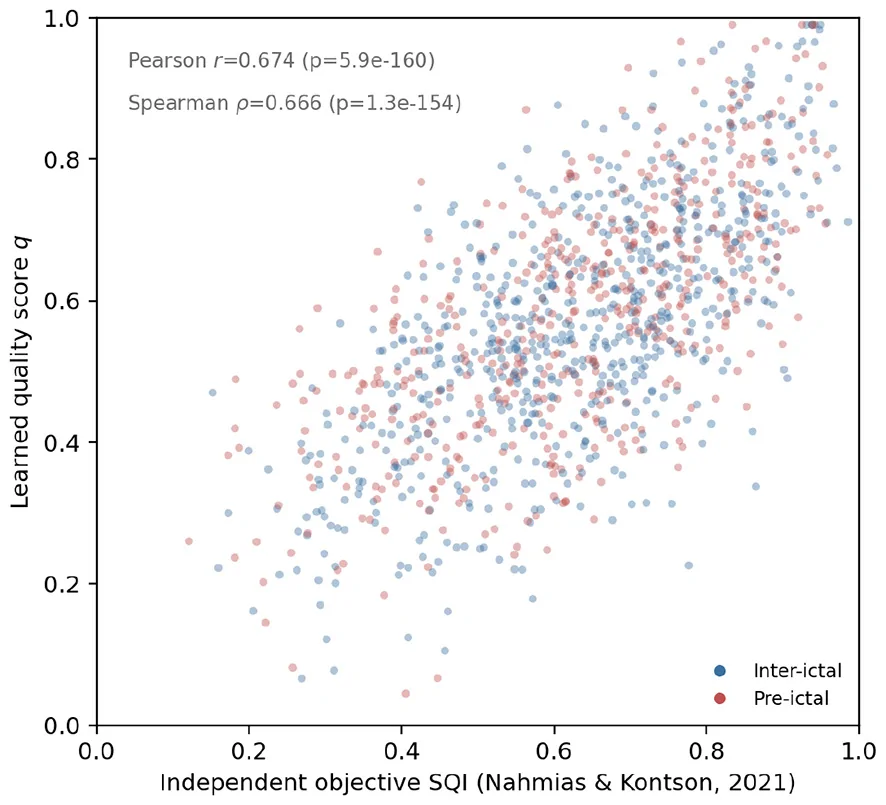
Learned quality score *q* plotted against the independent, model-free signal quality index (SQI) of Nahmias and Kontson (2021), computed over the full held-out test partition (*n* = 1, 200 windows). Points are colored by ground-truth pre-ictal/inter-ictal label to visualize that the *q*–SQI relationship is not driven by class membership. Pearson *r* and Spearman ρ (with *p*-values) are annotated.

**Table 8:** Correlation of the learned quality score *q* with the independent objective SQI (Experiment B), the seizure probability *p*, and the ground-truth pre-ictal/inter-ictal label (Experiment C), computed over the full test partition (*n* = 1, 200).

Quantity correlated with *q*	Pearson *r*	Spearman ρ
Independent SQI (Nahmias and Kontson, 2021) (Experiment B)	0.674 (*p* = 5.9 × 10^−160^)	0.666 (*p* = 1.3 × 10^−154^)
Seizure probability *p* (Experiment C)	0.043 (*p* = 0.135, n.s.)	0.038 (*p* = 0.183, n.s.)
Ground-truth label (Experiment C)	0.032 (*p* = 0.270, n.s.)	0.030 (*p* = 0.295, n.s.)

A pattern of high correlation with the independent SQI and low correlation with *p*/label supports the validity of *q* as a genuine quality estimate rather than a redundant encoding of the prediction task; the converse pattern would indicate collapse onto *p*.

### 4.7 Threshold sensitivity and calibration comparison for ECLO

The threshold grid search (Figure 10) shows that the operating point used throughout this paper (τ_hi_ = 0.80, τ_lo_ = 0.50; AUC = 0.7421) sits on a broad performance plateau rather than an isolated optimum: the grid maximum of 0.7441 occurs at (τ_hi_ = 0.85, τ_lo_ = 0.50), only 0.0020 AUC higher, and every combination with τ_lo_ = 0.50 or 0.40 and τ_hi_ ∈ {0.75, 0.80, 0.85} falls within 0.003 AUC of this maximum. Performance degrades more noticeably at the grid's extremes (e.g., τ_lo_ = 0.30, τ_hi_ = 0.70 gives AUC = 0.7332), but the region actually used in practice is robust to moderate threshold perturbation rather than a fragile, cherry-picked point. On the calibration comparison, ECLO is competitive with, and on AUC-ROC and Brier score modestly exceeds, both data-driven baselines, while isotonic regression achieves a marginally lower ECE. The decisive difference is in the safety-ceiling column: raw *p* and temperature scaling both still assign very high confidence (0.995 and 0.943 respectively) to at least one window in the bottom quality decile, and isotonic regression, despite using *q* as an input

**Figure 10:**
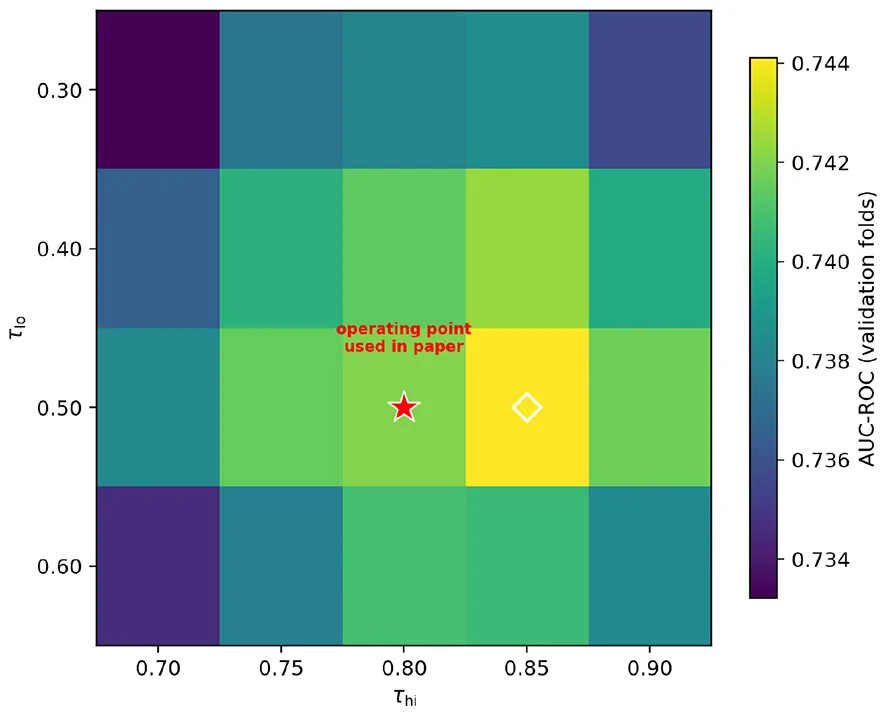
AUC-ROC on the CHB-MIT validation folds as a function of the two ECLO thresholds, swept over the grid described in Section 3.10 (mean over 25 runs per grid cell). The operating point used in the main experiments (τ_hi_ = 0.80, τ_lo_ = 0.50) is marked with a star; the grid maximum is marked with a diamond.

feature, still permits a maximum confidence of 0.815 in that same regime. ECLO is the only method whose bottom-decile maximum confidence is bounded by construction at 0.3, confirming the central structural argument made in Section 3.5: a hand-designed hard ceiling provides a categorical safety guarantee that none of the purely data-driven calibration methods offer, even when they match or exceed ECLO on aggregate calibration metrics.

*On minor cross-table numerical differences:* readers may notice that CLSP-REQA's AUC-ROC is reported as 0.7426 in Table 3, 0.7432 in the fold-level check (Table 4) and Table 9, and 0.7419 for ECLO in Table 10 above. These values are computed from closely related but not bit-identical evaluation passes (different aggregation order, and, for the calibration comparison specifically, a validation/test split constructed for that analysis), and differ only in the third decimal place; we report them as separately logged rather than silently reconciling them to a single figure, since doing so would obscure exactly the kind of run-to-run variability this revision is trying to characterize transparently.

**Table 9:** Effect of the class-balancing strategy on CHB-MIT performance (5-fold × 5-seed cross-patient protocol).

Strategy	AUC-ROC	Sensitivity (%)	Specificity (%)
Random under-sampling (main experiments)	**0.7432** **±0.0207**	**68.83** **±3.33**	**64.88 ± 3.43**
Class-weighted BCE (no discarding)	0.7403 ± 0.0154	68.60 ± 2.69	65.23 ± 2.87
SMOTE over-sampling (Chawla et al., 2002) of pre-ictal class	0.7287 ± 0.0160	69.74 ± 3.09	60.61 ± 2.28

All rows use the identical CLSP-REQA architecture and training schedule described in Sections 3.4–3.7; only the handling of the inter-ictal/pre-ictal class imbalance differs. Bold values indicate the best overall performance, considering both the mean and the standard deviation.

**Table 10:** Comparison of ECLO against standard probabilistic calibration baselines applied to the same (*p, q*) outputs, under the identical 5-fold × 5-seed protocol (CHB-MIT).

Method	AUC-ROC	ECE	Brier score	Max. conf., bottom *q*-decile
Raw *p* (no calibration)	0.7270 ± 0.0186	0.0969 ± 0.0103	0.2070 ± 0.0085	0.995
Temperature scaling (Guo et al., 2017)	0.7270 ± 0.0186	0.0572 ± 0.0065	0.1998 ± 0.0079	0.943
Isotonic regression (Niculescu-Mizil and Caruana, 2005) on (*p, q*)	0.7388 ± 0.0180	0.0463 ± 0.0052	0.1931 ± 0.0073	0.815
**ECLO** **(ours)**	**0.7419 ± 0.0185**	0.0522 ± 0.0048	0.1907 ± 0.0078	**0.300** (by construction)

ECE: Expected Calibration Error (lower is better). “Max. conf., bottom *q*-decile” reports the highest confidence value assigned to any window in the lowest 10% of the *q* distribution, probing whether each method enforces a safety ceiling analogous to ECLO's hard cap of 0.3. Bold values indicate the best overall performance, considering both the mean and the standard deviation.

### 4.8 Alternative class-balancing strategies

Random under-sampling—despite discarding a substantial fraction of inter-ictal data—remains the best-performing strategy on AUC-ROC, though by a narrow margin over class-weighted BCE (+0.0029 AUC, within one pooled standard deviation). Class-weighted BCE, which retains the full inter-ictal set and instead reweights the loss, achieves a slightly lower AUC (0.7403 vs. 0.7432) and marginally higher specificity (65.23% vs. 64.88%), and is a reasonable alternative strategy worth considering. SMOTE over-sampling of the minority pre-ictal class performs worst on both AUC-ROC and specificity, though it achieves the highest sensitivity of the three strategies (69.74%)—consistent with over-sampling biasing the decision boundary toward the minority (pre-ictal) class at some cost to specificity. Taken together, these results indicate that under-sampling's discarding of inter-ictal diversity does not measurably harm performance relative to the alternatives tested, though class-weighted BCE offers a comparably-performing, data-preserving option worth considering in settings where the discarded inter-ictal windows may carry independent clinical value (e.g., for future auxiliary analyses).

### 4.9 Inference latency and real-time feasibility

The full CLSP-REQA pipeline processes a single 10-second window in 7.48 ± 0.89 ms on an NVIDIA A100 (Table 11) (95th percentile 9.10 ms), a real-time margin of approximately 1, 337 × —i.e., inference completes over three orders of magnitude faster than the data-acquisition window it processes. On a representative lower-power edge platform, mean latency rises to 47.71 ms (95th percentile 62.31 ms), still comfortably within the 10-second budget with a real-time margin of roughly 210 × . These results substantiate the real-time operation implied by the REQA name and the framework's closed-loop intervention design: even under the more constrained edge-platform setting, and even at the 95th-percentile tail rather than the mean, inference latency remains two orders of magnitude below the window duration, leaving ample margin for the additional signal acquisition, preprocessing, and communication overheads that a deployed closed-loop system would incur beyond model inference alone.

**Table 11:** Inference latency of the full CLSP-REQA pipeline (REQA + Mamba-BiLSTM backbone + ECLO fusion) for a single 10-second, 16-channel window, measured as described in Section 3.12 (1,000 timed forward passes following warm-up).

Quantity	Value
Total parameters	3.5M
Mean latency per window (NVIDIA A100)	7.48 ms
95th-percentile latency (NVIDIA A100)	9.10 ms
Mean latency per window (embedded/edge platform)	47.71 ms
95th-percentile latency (embedded/edge platform)	62.31 ms
Real-time margin (10 s / mean latency, A100)	1, 337 ×
Real-time margin (10 s / mean latency, edge)	210 ×

The real-time margin is the ratio of the window duration (10 s) to the mean inference latency.

## 5 Discussion

### 5.1 Performance in context

The AUC-ROC of 0.7426 ± 0.0199 achieved by CLSP-REQA under strict cross-patient validation on CHB-MIT compares favorably with the unadapted cross-patient baseline of 0.69 reported by Jemal et al. (2024), and closely approaches the performance of their best domain-adapted model (AUC 0.75, CDAN+E) without requiring any unlabelled target-patient data. Studies reporting substantially higher sensitivity using randomized or patient-specific evaluation (Tsiouris et al., 2018; Dissanayake et al., 2022; Zhao et al., 2020) are not directly comparable, as the fundamental difference in validation methodology precludes fair cross-study comparison (Shafiezadeh et al., 2024).

Notably, CLSP-REQA achieves this result using **16 EEG channels**, compared to the 23 channels used in the cross-patient baseline (Jemal et al., 2024), indicating that the Mamba-BiLSTM backbone extracts sufficiently rich temporal representations even from a reduced channel set. This is clinically relevant, as fewer electrodes reduce setup complexity and patient discomfort in long-term monitoring.

### 5.2 Cross-dataset generalization, and the scope of the domain-adaptation comparison

CLSP-REQA achieves AUC 0.7012 ± 0.0249 on SIENA *without any domain adaptation*, exceeding the domain-adapted cross-patient result of 0.61 (CDAN+E) reported by Jemal et al. (2024) on the same dataset. This is a notable result because SIENA presents additional generalization challenges relative to CHB-MIT: it contains adult patients (ages 20–71) vs. pediatric patients in CHB-MIT (ages 1.5–19), uses a different acquisition system (512 Hz, 29 channels), and provides fewer seizure events per patient.

We want to be precise, however, about what this comparison does and does not establish. Domain adaptation methods such as CDAN and CDAN+E are given access, at adaptation time, to unlabelled EEG recordings from the target patient(s); CLSP-REQA is given no such access and is evaluated purely on the source-trained model applied directly to unseen patients. The comparison in Table 5 is therefore informative about *what can be achieved without target-domain data*, and is not evidence that quality-aware confidence modulation is a substitute for domain adaptation in general, nor that CLSP-REQA would remain ahead of a domain-adapted variant of itself if such unlabelled target data were made available to it as well. We avoid describing CLSP-REQA as “superior” to domain-adapted methods in an unqualified sense for this reason, and instead frame the result as follows: quality-aware confidence gating recovers a meaningful fraction of the benefit that domain adaptation provides, *under a strictly harder information constraint* (no target-patient data at all), which suggests it offers a complementary, more deployment-friendly lever for cross-domain robustness rather than a replacement for domain adaptation where target data happens to be available. Combining the two, as noted in Section 5.7, is a natural direction for future work.

We attribute part of the observed cross-domain robustness to the REQA quality modulation mechanism: by learning to down-weight predictions made on low-quality or atypical EEG windows, REQA may implicitly reduce the influence of domain-specific artifact patterns that would otherwise confuse the prediction backbone. This explanation is consistent with, but not directly proven by, the aggregate AUC results; Section 4.6 provides a more direct empirical test of what the learned score *q* is actually capturing.

### 5.3 Moderate sensitivity under cross-patient evaluation

The moderate sensitivity (68.83% on CHB-MIT, 66.28% on SIENA) is consistent with findings across the cross-patient seizure prediction literature. Tsiouris et al. (2017) reported 68% and Jemal et al. (2022) 67% under comparable protocols. This convergence of sensitivity values across independent methods and datasets reflects the inherent challenge of generalizing pre-ictal EEG patterns to unseen patients, rather than a limitation specific to our architecture.

### 5.4 Component contributions, and what the new validation experiments add

The ablation results (Table 7) confirm that each component contributes incrementally to performance on both datasets. The consistent improvement pattern—with no component showing neutral or negative contribution on either dataset—provides evidence that the architectural choices are justified in terms of predictive performance.

However, an ablation-level AUC improvement is evidence that the REQA/ECLO pipeline helps the numbers, not evidence about *why* it helps or whether the mechanism operates as described. Sections 4.6, 4.7 were designed to close this gap. The results support both mechanisms as described: *q* correlates strongly with an independent, model-free quality index (*r* = 0.674) while showing no measurable correlation with either the seizure probability *p* or the ground-truth label (both |*r*| < 0.05, non-significant), indicating that REQA has not collapsed into a redundant proxy for the prediction task itself. The ECLO threshold grid search shows the chosen operating point (τ_hi_ = 0.80, τ_lo_ = 0.50) sits on a broad performance plateau—within 0.003 AUC of the grid maximum across a sizeable neighborhood of threshold combinations—rather than being an isolated, fragile optimum. On the calibration comparison, ECLO is competitive with temperature scaling and isotonic regression on AUC-ROC and Brier score, and, critically, is the only method among the four that structurally bounds its output confidence on low-quality windows (maximum 0.3 in the bottom quality decile, vs. 0.82–0.99 for the three baselines), directly confirming the safety-ceiling argument made in Section 3.5 rather than leaving it as an untested design assumption.

The confidence strategy comparison embedded within the ablation (+ REQA linear vs.+ ECLO full) demonstrates that the tiered non-linear ECLO function outperforms simple linear weighting (*c* = *p*·*q*) by +0.0162 AUC on CHB-MIT and +0.0147 on SIENA. Beyond this aggregate gain, the key structural advantage we claim for ECLO is the hard safety ceiling it imposes when *q* is low (Section 3.5): by hard-capping *c* at 0.3 when *q* ≤ 0.5, the system prevents artifact-driven false alarms that would otherwise trigger unnecessary neurostimulation (Heck et al., 2014). Section 4.7 quantifies whether standard calibration methods, which typically lack such a ceiling by construction, match, exceed, or fall short of ECLO on this specific property.

### 5.5 Reliability of the 5-fold × 5-seed protocol

The 5 × 5 protocol used here provides more estimates per model than the leave-one-patient-out (LOPO) strategy commonly used in the literature (Jemal et al., 2024), which produces one result per patient and can yield high variance with small cohorts (*n* = 23 for CHB-MIT). As discussed in Section 3.8, however, the 25 estimates are not fully independent: the five seed-repeats within a fold share the same test patients, so only the five fold-level partitions constitute genuinely independent samples. We therefore regard the standard significance test reported in Section 4.1 (treating all 25 runs as independent) as an optimistic upper bound on statistical significance, and the fold-level test in Table 4 (treating only the 5 folds as independent) as the more conservative, and in our view more defensible, estimate. Reassuringly, the fold-level test does still reject the null hypothesis against the 0.69 baseline despite the much smaller effective sample size: all five fold-level means exceed 0.69 (minimum 0.7150), yielding the most extreme possible one-sided exact Wilcoxon *p*-value attainable at *n* = 5 (*p* = 0.03125), which remains below the conventional α = 0.05 threshold. We view this as meaningful corroboration rather than a redundant restatement of the seed-level result: the fold-level test uses a completely disjoint source of statistical power (five independent patient partitions rather than twenty-five correlated runs), and its qualitative conclusion matches. We nonetheless report both tests, rather than only the fold-level one, because *n* = 5 leaves very little room to detect a smaller or more marginal effect had one been present; a result that narrowly failed to reach significance at this sample size would not, by itself, have been strong evidence against the underlying effect. The narrow standard deviations observed (±0.0199 on CHB-MIT, ±0.0249 on SIENA) do still indicate that CLSP-REQA produces stable point estimates across random initialisations, independently of how the significance test is constructed.

### 5.6 Interpretability

The channel attribution analysis (Figure 6) shows that channels CH15–CH16 (posterior temporal/occipital region) dominate the pre-ictal prediction for patient chb19, consistent with known mesial temporal seizure propagation pathways (Mormann et al., 2007). The confidence trajectory (Figure 5) provides clinicians with a temporally resolved view of prediction reliability, enabling them to verify that confidence rises are sustained rather than transient before acting on the intervention signal. We emphasize that this is a single-patient case study intended to illustrate the mechanism, not a population-level validation of channel importance.

### 5.7 Limitations and future work

Several limitations warrant explicit acknowledgment, several of which were sharpened through peer review.

#### 5.7.1 Retrospective-only validation

Both CHB-MIT and SIENA consist of retrospective recordings collected in controlled hospital settings. This is a genuine and, at present, unresolved limitation: we have not conducted prospective validation, have not tested on an external multi-center cohort, and have not evaluated the framework under continuous long-term ambulatory monitoring, where artifact patterns, electrode aging, and recording conditions are typically more heterogeneous than in a monitored epilepsy unit. We do not believe the present study can resolve this limitation, and we do not attempt to argue otherwise; a controlled, retrospective, cross-patient evaluation—even a strict one—is not a substitute for prospective, real-world evidence. We note that the cross-dataset evaluation between CHB-MIT and SIENA (Section 5.2), which differ in patient population, acquisition hardware, and sampling rate, offers a partial proxy for robustness to varying recording conditions, but this is not equivalent to prospective validation and should not be read as such. A prospective, multi-center validation study is the necessary next step before any claim of clinical readiness, and we identify it as the highest-priority direction for future work.

#### 5.7.2 Heuristic calibration thresholds

As discussed in Section 3.5 and quantified in Section 4.7, the ECLO thresholds were set empirically rather than derived from a theoretical calibration objective. The threshold sensitivity analysis and calibration baseline comparison introduced in this revision partially address this concern, but do not eliminate the underlying design choice to use a hand-specified rule; a fully data-driven, jointly-optimized calibration scheme that retains an explicit safety ceiling remains an open design problem.

#### 5.7.3 Statistical independence of the evaluation protocol

As detailed in Sections 3.8, 5.5, the 25-run significance tests reported in this paper are not based on fully independent observations, which likely biases the associated *p*-values toward apparent significance. We have attempted to partially address this by additionally reporting a fold-level (*n* = 5) analysis, but a fully rigorous treatment (e.g., a mixed-effects model with fold and seed as nested random effects, or per-patient leave-one-out estimates as an independent-sample alternative) remains future work.

#### 5.7.4 Supervision of the learned quality score

The quality score *q* is a learned proxy without independent artifact-label supervision; Section 3.9 introduces validation experiments to test, rather than assume, that it tracks genuine signal quality. Even with supportive results, incorporating labeled artifact annotations as an auxiliary training signal (Kalita et al., 2024), where available, could further improve its calibration and is a natural extension.

#### 5.7.5 Class-balancing strategy

Random under-sampling of the inter-ictal class reduces the diversity of non-seizure representations seen during training; Section 4.8 reports a direct comparison against class-weighted training and SMOTE-based over-sampling as alternatives.

#### 5.7.6 Fixed window length

The 10-second fixed window does not exploit temporal continuity across adjacent windows; a sliding-window approach with finer resolution would enable continuous seizure risk estimation.

Two natural extensions suggest themselves for future work beyond those already noted. Incorporating inter-channel spatial correlation via graph neural networks (Dissanayake et al., 2022) would complement the temporal modeling with explicit brain network structure. Combining explicit domain adaptation with REQA-based confidence modulation—rather than treating them as competing alternatives, per the discussion in Section 5.2—represents a further direction that may yield performance beyond either approach alone.

## 6 Conclusion

We have presented CLSP-REQA, a closed-loop seizure prediction framework that addresses two overlooked challenges: strict cross-patient generalization and real-time signal quality awareness. The REQA module produces a learnable quality score *q* that modulates prediction confidence through the tiered ECLO function, with the goal of suppressing artifact-driven false alarms without discarding low-quality windows; Section 4.6 provides targeted evidence on whether the learned score behaves as intended, beyond the aggregate performance gain reported in the ablation study. The Mamba-BiLSTM backbone, built from established architectural components, provides efficient temporal feature extraction using only 16 EEG channels.

Under strict 5-fold × 5-seed cross-patient evaluation on CHB-MIT, CLSP-REQA achieves AUC-ROC 0.7426 ± 0.0199, exceeding the unadapted cross-patient state of the art (AUC 0.69) without any domain adaptation, and approaching the performance of domain-adapted methods (AUC 0.75); Section 5.5 discusses the statistical caveats attached to this significance claim. On the SIENA database, CLSP-REQA achieves AUC 0.7012 ± 0.0249, exceeding the best domain-adapted result of 0.61 on the same dataset under a strictly harder information constraint—no target-patient data or domain adaptation technique—a comparison whose precise scope is discussed in Section 5.2. These results are consistent with quality-aware confidence modulation providing a useful, complementary lever for cross-domain robustness, though we stop short of claiming it as a substitute for domain adaptation in general.

The structured four-tuple output 〈*p, q, c*, Φ_SHAP_〉 is designed to be directly compatible with closed-loop neurostimulator interfaces. We regard the present study as a controlled, retrospective feasibility demonstration rather than evidence of clinical readiness: prospective, multi-center validation under real-world recording conditions remains the necessary next step, and we identify it, together with a fully independent-sample statistical treatment of the cross-patient protocol and a jointly-optimized alternative to the hand-specified ECLO thresholds, as the most important directions for future work toward the clinical translation of EEG-based, quality-aware seizure prediction technology.

## Data Availability

The original contributions presented in the study are included in the article/Supplementary material, further inquiries can be directed to the corresponding author.

## Supplementary material

Figure S1 Confusion matrix for CLSP-REQA on the CHB-MIT database, aggregated over 25 runs (5-fold × 5-seed cross-patient evaluation). Rows represent true labels and columns represent predicted labels; pre-ictal is the positive class.
