# Supplementary material for: CLSP-REQA: a real-time quality-aware closed-loop seizure prediction framework with Mamba-BiLSTM and confidence-gated intervention
Source: Front Neuroinform. 2026 Sep 30;20:1888743. doi: 10.3389/fninf.2026.1888743 (PMC13661927; doi:10.3389/fninf.2026.1888743)
Supplement: Supplementary file 1 [file Data_Sheet_1.pdf]

Supplementary Material

CLSP-REQA: A Real-Time Quality-Aware Closed-Loop Seizure Prediction Framework with Mamba-BiLSTM and Confidence-Gated Intervention

Fig. S1: Confusion Matrix

Fig. S1 presents the aggregated confusion matrix for CLSP-REQA on the CHB-MIT database, computed over all 25 independent runs (5-fold × 5-seed cross-patient evaluation).

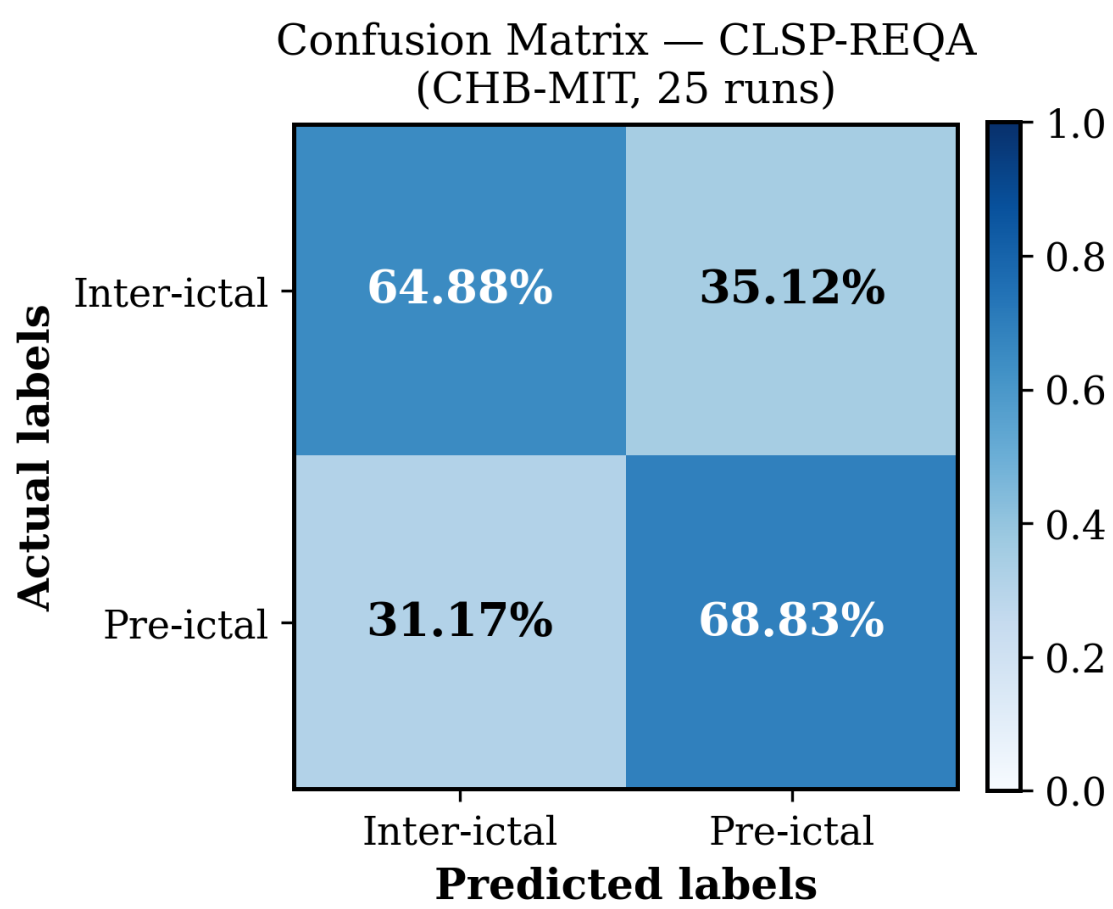

Figure S1: Confusion matrix for CLSP-REQA on CHB-MIT, aggregated over 25 runs. Rows represent true labels; columns represent predicted labels. Pre-ictal: positive class; Inter-ictal: negative class.
